# Supplementary material for: Metasurface‐Programmable Wireless Network‐On‐Chip
Source: Adv Sci (Weinh). 2022 Jun 24;9(26):2201458. doi: 10.1002/advs.202201458 (PMC9475537; doi:10.1002/advs.202201458)
Supplement: Supplementary file 1 — Supporting Information [file ADVS-9-2201458-s001.pdf]

ADVANCED SCIENCE
Open Access

Supporting Information

for *Adv. Sci.*, DOI 10.1002/advs.202201458

Metasurface-Programmable Wireless Network-On-Chip

*Mohammadreza F. Imani, Sergi Abadal and Philipp del Hougne**

Supporting Information

Metasurface-Programmable Wireless Network-on-Chip

*Mohammadreza F. Imani, Sergi Abadal, and Philipp del Hougne**

Additional Characterizations of the Programmable Metasurfaces

In this supplemental note, we provide further analysis and characterization of the programmable metasurfaces that we consider in the main text.

Remark S1: The main contributions of our work, namely proposal and analysis of the *in situ* reprogrammable shaping of on-chip wireless channels to suppress inter-symbol interference, are independent of the specific choice of the metasurface design. The designs that we utilize are illustrative of suitable designs but not heavily optimized.

Our goal in this supplemental note is to shed further light on the resonant behavior of the programmable meta-atoms. In the main text, we characterized

(i) an infinite array of the programmable meta-atom under normal plane wave incidence (i.e., periodic boundary conditions) in Figure 3a,b, and

(ii) the field fluctuations that a 5×5 array of 2×2 meta-atoms can induce *in situ* in the targeted complex scattering setup in Figure 3c,d.

While the former is a standard characterization technique, it does not provide insightful information about the operating bandwidth because it considers a scenario that is too different from our actual deployment scenario. On the other hand, the latter is such a deployment-specific metric that it does not clearly reveal resonant features of the meta-atoms.

Therefore, in this supplemental note, we study the electrical energy stored inside the volume of the meta-atoms and meta-pixels discussed in the main text. Resonances are known to enhance wave-matter interactions because the dwell time of a wave is strongly increased at resonance, resulting in significantly more energy being stored inside resonant volumes. The relation between resonance, dwell time, and stored energy is intuitive but also well-established and formalized in mesoscopic wave physics[1–3]. Specifically, we integrate the electric field's intensity over the volume of the considered meta-atom (proportionality constants are neglected

for simplicity in the following) for a given illumination. This stored-energy metric will therefore clearly indicate resonant features.

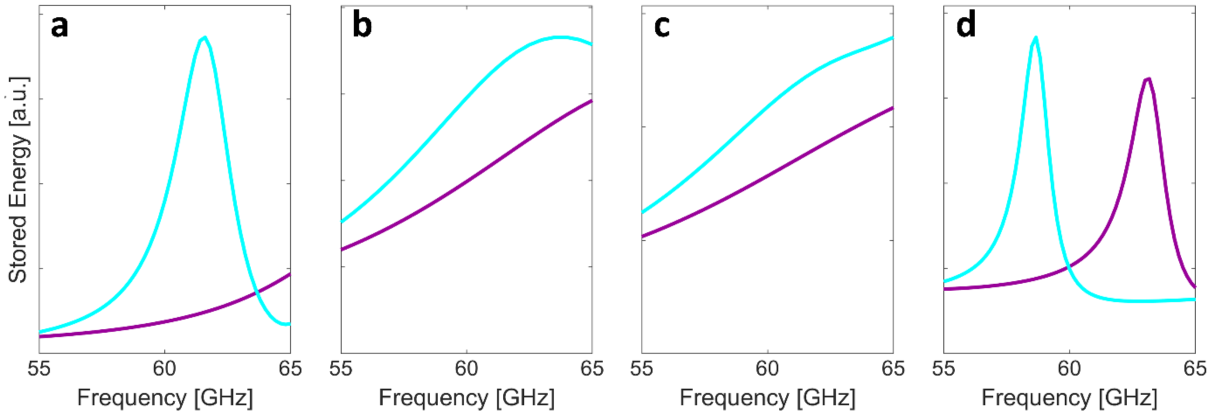

Figure S1. Stored energy. a) Single meta-atom in periodic array (similar to Figure 3a,b in the main text) under normal plane wave illumination. b) Single meta-pixel (i.e., 2×2 array of meta-atoms) in free-space setup (see explanations below) under normal plane wave illumination. c) Single meta-pixel (i.e., 2×2 array of meta-atoms) in free-space setup (see explanations below) under oblique (30°) plane wave illumination. d) Single meta-atom of the alternative design in free-space setup (see explanations below) under normal plane wave illumination. Note that the proportionality constant of the arbitary units of stored energy is different in each subfigure, but all vertical axes start at 0.

To start, in Figure S1a we consider the single meta-atom in the periodic array setup under normal plane wave illumination from Figure 3a,b. A clear resonant feature close to the center of the operating band is seen for one of the two chosen states. At resonance, the meta-atom stores significantly more energy.

Next, instead of considering a periodic array setup, we consider a free-space setup. Therein, a single meta-pixel (i.e., a 2×2 array of meta-atoms) together with the chip package's metallic ceiling is considered in "free space" – emulated by an absorbing boundary condition (referred to as "radiation boundary" in Ansys HFSS). In Figure S1b we see that the stored energy as a function of frequency is very different in this setting, but a significant difference between the two considered states persists. Indeed, the metallic ceiling and its coupling to the meta-atoms determine in a highly non-trivial way the amount of energy coupled into the meta-atom. Moreover, the angle of incidence affects the efficiency with which the resonance can be excited. At oblique (30°) instead of normal incidence, the curves are markedly different – see Figure S1c. This dependence on the angle of incidence originates from the fact that the wave impedance of the incident wave depends on the angle of incidence, and thereby the reflection and transmission coefficients between free space and the meta-pixel, too.

In the targeted deployment scenario, that is, inside the chip enclosure, waves with all possible angles of incidence impinge on the meta-pixels, resulting in a highly complicated excitation. This complexity cannot be captured by analyzing the reflection coefficient for one or a few

angles of incidence, which motivates the *in situ* characterization reported in Figure 3c,d. Whereas metasurfaces for free-space applications are fully characterized in terms of a single excitation channel (usually normal incidence), in a few cases also for a few oblique-incidence excitation channels, in rich-scattering applications like WNoCs such characterizations provide an incomplete picture because many excitation channels relevant to rich-scattering are neglected.

For completeness, we also plot the stored energy for the two considered states of the alternative meta-atom design in Figure S1d. While clear resonant features are seen, the *in situ* characterization yields a much more complex response – see Figure 7. This observation underlines once again the importance of an *in situ* characterization that accounts for the complexity of the targeted application scenario.

Finally, we also plot the field magnitude at a few selected frequencies within the programmable meta-atoms as observed *in situ* (in the chip environment). In Figure S2 it is evident that the meta-pixels are not all excited in the same manner, because their excitation depends in a highly complicated way on the irregular scattering within the chip enclosure. Even proximity to the transmitter does not result in a markedly stronger excitation because the latter depends on the angle of incidence and interferences with other waves reflected inside the enclosure.

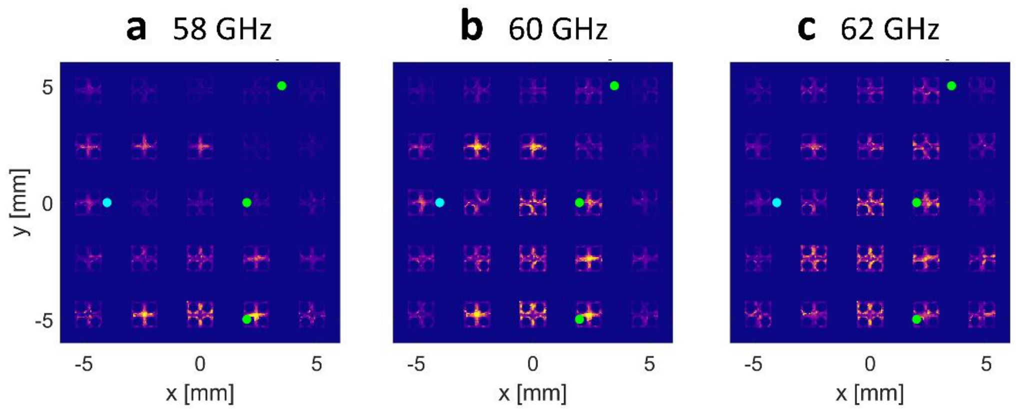

Figure S2. Spatial distribution of the field component that is perpendicular to the chip plane, evaluated *in situ* at $h = 920$ µm, i.e., inside the meta-atoms. We plot the field intensity at three frequencies using the same color scale as in Figure 1a-c.

Supplementary References

[1] S. Rotter, S. Gigan, *Rev. Mod. Phys.* **2017**, *89*, 015005.
[2] M. Durand, S. M. Popoff, R. Carminati, A. Goetschy, *Phys. Rev. Lett.* **2019**, *123*, 243901.
[3] P. del Hougne, R. Sobry, O. Legrand, F. Mortessagne, U. Kuhl, M. Davy, *Laser Photonics Rev.* **2021**, 2000335.
